# Supplementary material for: Hybrid Models and Biological Model Reduction with PyDSTool
Source: PLoS Comput Biol. 2012 Aug 9;8(8):e1002628. doi: 10.1371/journal.pcbi.1002628 (PMC3415397; doi:10.1371/journal.pcbi.1002628)
Supplement: Text S4 — Complete source code for the PyDSTool package (version 0.88.120504). Includes API documentation and help files linking to web pages. This file is identical to the current public release on Sourceforge.net. (ZIP) [file pcbi.1002628.s004.zip › PyDSTool/html/PyDSTool.Trajectory'-module.html]

xml version="1.0" encoding="ascii"?


PyDSTool.Trajectory'


| Home | Trees | Indices | Help | PyDSTool |
| --- | --- | --- | --- | --- |

Package PyDSTool ::
Module Trajectory'

[hide private]

[frames] | no frames]

# Module Trajectory'

source code

Trajectory classes.

Robert Clewley, June 2005.


|  |  |
| --- | --- |
| Classes | [hide private] |

HybridTrajectory  
Hybrid, parameterized, trajectory class.

Trajectory  
Parameterized and non-parameterized trajectory class.


|  |  |
| --- | --- |
| Functions | [hide private] |

|  |  |
| --- | --- |
| convert\_ptlabel\_events(pts)  Creates an eventTimes-like dictionary from a pointset's labels. | source code |

|  |  |
| --- | --- |
| findApproxPeriod(traj, t0, t1\_guess=None, T\_guess=None, coordname=None, ttol=1e-05, rtol=0.01, guess\_tol=0.1)  findPeriod does not ensure minimum period (it is up to the user to select a good initial guess, but guess\_tol fraction of T\_guess will be checked as a bracketing interval for bisection. | source code |

|  |  |
| --- | --- |
| numeric\_to\_traj(vals, trajname, coordnames, indepvar=None, indepvarname=`'``t``'`, indepdomain=None, all\_types\_float=True, discrete=True)  Utility function to convert one or more numeric type to a Trajectory. | source code |

|  |  |
| --- | --- |
| pointset\_to\_traj(pts)  Convert a pointset into a trajectory using linear interpolation, retaining parameterization by an independent variable if present. | source code |


|  |  |
| --- | --- |
| Variables | [hide private] |

API = `API_class()`

Continuous = `Continuous Domain`

Discrete = `Discrete Domain`

Inf = `inf`

LargestInt32 = `2147483647`

NAMESEP = `'.'`

ONES = `['1', '1.0', '1.', '(1)', '(1.0)', '(1.)']`

ZEROS = `['0', '0.0', '0.', '(0)', '(0.0)', '(0.)']`

\_1DimplicitSolveMethods = `['newton', 'bisect', 'steffe']`

\_all\_complex = `(<type 'complex'>, <type 'numpy.complexfloating...`

\_all\_float = `(<type 'float'>, <type 'numpy.floating'>, <type '...`

\_all\_int = `(<type 'int'>, <type 'numpy.integer'>, <type 'numpy...`

\_all\_numpy\_complex = `(<type 'numpy.complex128'>, <type 'numpy....`

\_all\_numpy\_float = `(<type 'numpy.float64'>, <type 'numpy.float...`

\_all\_numpy\_int = `(<type 'numpy.int32'>, <type 'numpy.int32'>, ...`

\_complex\_types = `(<type 'complex'>, <type 'numpy.complexfloati...`

\_float\_types = `(<type 'float'>, <type 'numpy.floating'>)`

\_implicitSolveMethods = `['newton', 'bisect', 'steffe', 'fsolve']`

\_indentstr = `' '`

\_int\_types = `(<type 'int'>, <type 'numpy.integer'>)`

\_num\_equivtype = `{<type 'float'>: <type 'numpy.float64'>, <typ...`

\_num\_maxmin = `{<type 'numpy.int32'>: [-2147483648, 2147483647]...`

\_num\_name2equivtypes = `{'float': (<type 'float'>, <type 'numpy...`

\_num\_name2type = `{'float': <type 'numpy.float64'>, 'int': <typ...`

\_num\_type2name = `{<type 'float'>: 'float', <type 'int'>: 'int'...`

\_num\_types = `(<type 'float'>, <type 'int'>, <type 'numpy.float...`

\_pytypefromtype = `{<type 'numpy.int32'>: <type 'int'>, <type '...`

\_real\_types = `(<type 'int'>, <type 'numpy.integer'>, <type 'fl...`

\_seq\_types = `(<type 'list'>, <type 'tuple'>, <type 'numpy.ndar...`

builtinFnSigInfo = `{'for': 4, 'getbound': 2, 'getindex': 1, 'g...`

builtin\_auxnames = `['globalindepvar', 'initcond', 'heav', 'if'...`

contained = `contained`

name\_chars\_RE = `re.compile(r'\w')`

notcontained = `notcontained`

null\_predicate = `null_predicate_class(None)`

num\_chars = `['0', '1', '2', '3', '4', '5', '6', '7', '8', '9']`

protected\_allnames = `['acos', 'asin', 'atan', 'atan2', 'ceil',...`

protected\_auxnamesDB = `ModelSpec internal helper class: auxfnD...`

protected\_macronames = `['for', 'if', 'max', 'min', 'sum']`

protected\_mathnames = `['acos', 'asin', 'atan', 'atan2', 'ceil'...`

protected\_randomnames = `['BPF', 'LOG4', 'NV_MAGICCONST', 'RECI...`

protected\_scipynames = `['sign', 'mod']`

protected\_specialfns = `['special_airy', 'special_airye', 'spec...`

syms = `{0: 'ENDMARKER', 1: 'NAME', 2: 'NUMBER', 3: 'STRING', 4...`

targetLangs = `['c', 'python', 'matlab']`

uncertain = `uncertain`


|  |  |
| --- | --- |
| Function Details | [hide private] |

|  |  |
| --- | --- |
| convert\_ptlabel\_events(pts) | source code |

Creates an eventTimes-like dictionary from a pointset's labels. (Event
X time recorded in a label is recorded as "Event:X")

|  |  |
| --- | --- |
| numeric\_to\_traj(vals, trajname, coordnames, indepvar=None, indepvarname=`'``t``'`, indepdomain=None, all\_types\_float=True, discrete=True) | source code |

Utility function to convert one or more numeric type to a Trajectory.
Option to make the trajectory parameterized or not, by pasing an indepvar
value.

To use integer types for trajectory coordinates unset the default
all\_types\_float=True argument.

To create interpolated (continuously defined) trajectories, set
discrete=False, otherwise leave at its default value of True.

  


|  |  |
| --- | --- |
| Variables Details | [hide private] |

### \_all\_complex

Value:
:   ```
    (<type 'complex'>,
     <type 'numpy.complexfloating'>,
     <type 'numpy.complex128'>,
     <type 'numpy.complex64'>,
     <type 'numpy.complex128'>)
    ```

### \_all\_float

Value:
:   ```
    (<type 'float'>,
     <type 'numpy.floating'>,
     <type 'numpy.float64'>,
     <type 'numpy.float32'>,
     <type 'numpy.float64'>)
    ```

### \_all\_int

Value:
:   ```
    (<type 'int'>,
     <type 'numpy.integer'>,
     <type 'numpy.int32'>,
     <type 'numpy.int32'>,
     <type 'numpy.int8'>,
     <type 'numpy.int16'>,
     <type 'numpy.int32'>,
     <type 'numpy.int64'>)
    ```

### \_all\_numpy\_complex

Value:
:   ```
    (<type 'numpy.complex128'>,
     <type 'numpy.complex64'>,
     <type 'numpy.complex128'>)
    ```

### \_all\_numpy\_float

Value:
:   ```
    (<type 'numpy.float64'>,
     <type 'numpy.float32'>,
     <type 'numpy.float64'>)
    ```

### \_all\_numpy\_int

Value:
:   ```
    (<type 'numpy.int32'>,
     <type 'numpy.int32'>,
     <type 'numpy.int8'>,
     <type 'numpy.int16'>,
     <type 'numpy.int32'>,
     <type 'numpy.int64'>)
    ```

### \_complex\_types

Value:
:   ```
    (<type 'complex'>, <type 'numpy.complexfloating'>)
    ```

### \_num\_equivtype

Value:
:   ```
    {<type 'float'>: <type 'numpy.float64'>,
     <type 'int'>: <type 'numpy.int32'>,
     <type 'numpy.integer'>: <type 'numpy.int32'>,
     <type 'numpy.floating'>: <type 'numpy.float64'>,
     <type 'numpy.int8'>: <type 'numpy.int32'>,
     <type 'numpy.int16'>: <type 'numpy.int32'>,
     <type 'numpy.int32'>: <type 'numpy.int32'>,
     <type 'numpy.int32'>: <type 'numpy.int32'>,
    ...
    ```

### \_num\_maxmin

Value:
:   ```
    {<type 'numpy.int32'>: [-2147483648, 2147483647],
     <type 'numpy.float64'>: [-inf, inf]}
    ```

### \_num\_name2equivtypes

Value:
:   ```
    {'float': (<type 'float'>,
               <type 'numpy.floating'>,
               <type 'numpy.float64'>,
               <type 'numpy.float32'>,
               <type 'numpy.float64'>),
     'int': (<type 'int'>,
             <type 'numpy.integer'>,
             <type 'numpy.int32'>,
    ...
    ```

### \_num\_name2type

Value:
:   ```
    {'float': <type 'numpy.float64'>, 'int': <type 'numpy.int32'>}
    ```

### \_num\_type2name

Value:
:   ```
    {<type 'float'>: 'float',
     <type 'int'>: 'int',
     <type 'numpy.integer'>: 'int',
     <type 'numpy.floating'>: 'float',
     <type 'numpy.int8'>: 'int',
     <type 'numpy.int16'>: 'int',
     <type 'numpy.int32'>: 'int',
     <type 'numpy.int32'>: 'int',
    ...
    ```

### \_num\_types

Value:
:   ```
    (<type 'float'>,
     <type 'int'>,
     <type 'numpy.floating'>,
     <type 'numpy.integer'>)
    ```

### \_pytypefromtype

Value:
:   ```
    {<type 'numpy.int32'>: <type 'int'>,
     <type 'numpy.float64'>: <type 'float'>}
    ```

### \_real\_types

Value:
:   ```
    (<type 'int'>,
     <type 'numpy.integer'>,
     <type 'float'>,
     <type 'numpy.floating'>)
    ```

### \_seq\_types

Value:
:   ```
    (<type 'list'>, <type 'tuple'>, <type 'numpy.ndarray'>)
    ```

### builtinFnSigInfo

Value:
:   ```
    {'for': 4,
     'getbound': 2,
     'getindex': 1,
     'globalindepvar': 1,
     'heav': 1,
     'if': 3,
     'initcond': 1,
     'max': 1,
    ...
    ```

### builtin\_auxnames

Value:
:   ```
    ['globalindepvar', 'initcond', 'heav', 'if', 'getindex', 'getbound']
    ```

### protected\_allnames

Value:
:   ```
    ['acos',
     'asin',
     'atan',
     'atan2',
     'ceil',
     'cos',
     'cosh',
     'degrees',
    ...
    ```

### protected\_auxnamesDB

Value:
:   ```
    ModelSpec internal helper class: auxfnDBclass object
    ```

### protected\_mathnames

Value:
:   ```
    ['acos',
     'asin',
     'atan',
     'atan2',
     'ceil',
     'cos',
     'cosh',
     'degrees',
    ...
    ```

### protected\_randomnames

Value:
:   ```
    ['BPF',
     'LOG4',
     'NV_MAGICCONST',
     'RECIP_BPF',
     'Random',
     'SG_MAGICCONST',
     'SystemRandom',
     'TWOPI',
    ...
    ```

### protected\_specialfns

Value:
:   ```
    ['special_airy',
     'special_airye',
     'special_ai_zeros',
     'special_bi_zeros',
     'special_ellipj',
     'special_ellipk',
     'special_ellipkinc',
     'special_ellipe',
    ...
    ```

### syms

Value:
:   ```
    {0: 'ENDMARKER',
     1: 'NAME',
     2: 'NUMBER',
     3: 'STRING',
     4: 'NEWLINE',
     5: 'INDENT',
     6: 'DEDENT',
     7: 'LPAR',
    ...
    ```

  


| Home | Trees | Indices | Help | PyDSTool |
| --- | --- | --- | --- | --- |

|  |  |
| --- | --- |
| Generated by Epydoc 3.0.1 on Fri May 4 15:24:05 2012 | http://epydoc.sourceforge.net |
